# Fellow-Workers and the Roll of Honour

**Published:** 1914-11-21

**Authors:** 


### ROUND THE WORLD.

[The Editor Invites Early Information and Contributions Marked "Fellow-Workers."]

### Fellow-Workers and the Roll of Honour.

MR. HENRY RUTHERFURD, M.B., C.M. Glasg., F.F.P.S. Glasg., lieutenant-colonel in the 3rd Scottish General Hospital, T.F., is surgeon to the Glasgow Royal Infirmary and examiner in surgery to the Universities of Glasgow and St. Andrews. Having pursued his technical studies at both Glasgow and Vienna Mr. Rutherfurd qualified in 1884, and subsequently held the posts of resident assistant surgeon and resident assistant physician to the Western Infirmary, Glasgow, house surgeon to the Royal Infirmary, Dundee, extra surgeon to the Royal Hospital for Sick Children, and extra dispensary surgeon to the Western Infirmary.

DR. M. JOHN COWAN, M.D., F.F.P.S. Glasg., D.Sc. Glasg., major in the same establishment, is professor of medicine in Anderson's College, Glasgow, and physician to the Royal Infirmary, as well as lecturer on clinical medicine to the University. A student of both Cambridge and Glasgow, Dr. Cowan qualified M.B., B.C. Cantab., in 1895. He has had previous military experience, having been physician to the Scottish National Red Cross Hospital in the South African War.

THE services of the honorary medical and surgical staff of the Leicester Royal Infirmary have been very heavily drawn upon in connection with the war. The entire surgical staff have been mobilised for the work of the 5th Northern General Hospital in the persons of Major Blakesley, Major Cecil Marriott, Major J. S. Sloane, Captain Bolton Carter, Captain Cumberlidge, and Captain Clare. The medical staff of the same institution has supplied Lieut.-Col. R. Proth, Major Sevestre, and Major Crosby, in addition to Colonel Astley V. Clarke, who has assumed the important duties of assistant director of medical services, North Midland Division. Six members of the resident medical staff have volunteered their services to the Government, and have been appointed to various branches of Imperial Service.

MAJOR HENRY JOHN BLAKESLEY is senior surgeon to the Leicester Royal Infirmary and surgeon to the Wyggeston Hospital, as well as surgeon to the Leicester and Rutland Lunatic Asylum. Formerly a student of Birmingham and University College Hospital, London, he qualified in 1881, subsequently becoming an F.R.C.S. Eng. Settling in Leicester, Major Blakesley became the holder of various responsible appointments, and identified himself with the interests of his fellow-practitioners there, particularly in regard to the Leicester Medical Society and the local division of the British Medical Association, over both of which he at one time presided.

MAJOR CECIL EDWARD MARRIOTT, M.B., M.C. Cantab., F.R.C.S. Eng., is also a student of University College Hospital, where he proceeded after preliminary studies at Cambridge. He is also a member of the surgical staff of the Leicester Royal Infirmary, where he was at one time house surgeon. In his younger days, Major Marriott held several resident posts at University College Hospital, whilst his present appointments include those of surgeon to the Children's Hospital at Leicester, and honorary medical referee to the Leamington Home for Incurables.

MAJOR JOHN STRETTON SLOANE, M.B., B.S. Lond., F.R.C.S. Eng., is likewise surgeon to the Leicester Royal Infirmary. As a "Bart.'s" student Major Sloane did very well in his final and class examinations, scoring honours at both the M.B. and B.S. Lond. examinations; whilst at his hospital he also carried off the senior entrance scholarship, the Blackenbury scholarship in surgery, and the Lawrence scholarship. In earlier years he was assistant surgeon to the Metropolitan Hospital and senior assistant to the throat department at St. Bartholomew's. Before qualifying Major Sloane also managed to find time to take the B.Sc. with honours.

CAPTAIN FELIX BOLTON CARTER, M.D., M.S. Lond., F.R.C.S. Eng., is a surgical colleague of the aforementioned at the Leicester Royal Infirmary, where he was house surgeon some years ago. Qualifying from University College Hospital, Captain Bolton Carter was awarded first-class honours at the B.S. Lond. in 1901, and before settling in practice held amongst other appointments those of senior house surgeon to the Royal Infirmary, Halifax, senior obstetric assistant, house physician, and house surgeon at University College Hospital.

CAPTAIN WILLIAM ISAAC CUMBERLIDGE, M.B., F.R.C.S. Eng., is junior to his colleagues at the Leicester Royal Infirmary, where he holds the post of assistant surgeon. As a student he won the natural science scholarships both at Cambridge and at St. Bartholomew's. Before taking up work at Leicester Captain Cumberlidge was house surgeon at "Bart.'s" and casualty officer to the London Temperance Hospital.

IT is well known that the Admiralty has secured the services of a number of eminent consultants as temporary consulting physicians and surgeons. Of these Sir William Watson Cheyne, Mr. Arthur Edmunds, Mr. C. M. Hughes, and Mr. G. R. Turner are attached to the Naval Hospital at Chatham.

SIR WILLIAM WATSON CHEYNE is, of course, one of the famous surgeons of the day and was created a baronet for his public services six years ago. Professor of clinical surgery at King's College Hospital, and honorary surgeon-in-ordinary to His Majesty, Sir William Watson Cheyne has for many years enjoyed a prominent position both as consultant and teacher, his original work having brought him various academic distinctions, including the F.R.S., the Jacksonian prize of the Royal College of Surgeons, and the Astley Cooper prize. He was decorated for his services as consulting surgeon to the Forces in South Africa at the time of the Boer War.

MR. ARTHUR EDMUNDS, M.B., B.S. Lond., F.R.C.S. Eng., is a member of the surgical staff at King's College Hospital, where he is also lecturer on surgical pathology, and where in his early days he had a distinguished academic career, his honours then including various scholarships, a gold medal in physiology, and the gold medal at the B.S. Lond. Formerly Mr. Edmunds was surgeon to out-patients at the Paddington Green Children's Hospital.

